# Supplementary material for: Earlier preterm birth is associated with a worse neurocognitive outcome in a rabbit model
Source: PLoS One. 2021 Jan 27;16(1):e0246008. doi: 10.1371/journal.pone.0246008 (PMC7840009; doi:10.1371/journal.pone.0246008)
Supplement: S1 Table — PCA31d n = 4, PCA30d n = 4, PCA29d n = 5, PCA28d n = 6 and PCA27d n = 2. Data displayed as mean and SD with significance compared to the term birth group indicated as * 0.05 ≥ p > 0.01; ** 0.01 ≥ p > 0.001; ***p < 0.001. (DOCX) [file pone.0246008.s001.docx]

|  | Term birth  PCA31 | Preterm  PCA30d | Preterm  PCA29d | Preterm  PCA28d | Preterm  PCA27d |
| --- | --- | --- | --- | --- | --- |
| Maternal weight | 5.1 ± 0.4 | 4.8 ± 0.4 | 4.9 ± 0.6 | 4.7 ± 0.5 | 4.1 ± 0.7 |
| Heart rate | 138 ± 11 | 145 ± 18 | 134 ± 14 | 156 ± 28 | 151 ± 10 |
| Saturation | 96 ± 1.4 | 96 ± 1.7 | 93 ± 4.9 | 94 ± 3.3 | 93 ± 2.3 |
| Temperature | 38.4 ± 0.5 | 38.2 ± 0.4 | 38.5 ± 0.3 | 38.5 ± 0.5 | 37.8 ± 0.3 |
| Litter size | 10 ± 1.5 | 11 ± 1.8 | 9 ± 2.9 | 11 ± 2.1 | 9 ± 1.1 |
| Stillbirth rate | 5.2 ± 6.0 | 6.0 ± 7.1 | 15.8 ± 11.3 | 13.8 ± 12.8 | 71.1 ± 5.5*** |

**S1 Table. Maternal observation at delivery and litter outcomes.** PCA31d n=4, PCA30d n= 4, PCA29d n= 5, PCA28d n = 6 and PCA27d n=2. Data displayed as mean and SD with significance compared to the term birth group indicated as * 0.05 ≥ p > 0.01; ** 0.01 ≥ p > 0.001; ***p < 0.001.
