# Supplementary material for: Earlier preterm birth is associated with a worse neurocognitive outcome in a rabbit model
Source: PLoS One. 2021 Jan 27;16(1):e0246008. doi: 10.1371/journal.pone.0246008 (PMC7840009; doi:10.1371/journal.pone.0246008)
Supplement: S2 Table — PCA31d n = 11, PCA30d n = 11, PCA29d n = 8 and PCA28d n = 11. Data displayed as mean and SD with significance compared to the term birth group indicated as * 0.05 ≥ p > 0.01; ** 0.01 ≥ p > 0.001; ***p < 0.001. (DOCX) [file pone.0246008.s002.docx]

|  | Term birth  PCA31 | Preterm  PCA30d | Preterm  PCA29d | Preterm  PCA28d |
| --- | --- | --- | --- | --- |
| Neuromotor Assessment | | | | |
| - Posture | 3.0 ± 0.0 | 3.0 ± 0.0 | 3.0 ± 0.0 | 2.8 ± 0.4 |
| - Gait | 3.6 ± 0.5 | 3.5 ± 0.6 | 2.6 ± 0.5** | 2.0 ± 0.6** |
| - Locomotion | 2.6 ± 0.5 | 2.4 ± 0.5 | 1.9 ± 0.4* | 1.4 ± 0.5** |
| - Motor Activity Fore Limb | 3.0 ± 0.0 | 3.0 ± 0.0 | 3.0 ± 0.0 | 3.0 ± 0.0 |
| - Motor Activity Hind Limb | 3.0 ± 0.0 | 3.0 ± 0.0 | 2.4 ± 0.5 | 2.2 ± 0.4 |
| - Motor Activity Head | 3.0 ± 0.0 | 2.8 ± 0.4 | 2.0 ± 0.0** | 1.8 ± 0.4** |
| - Activity Duration | 3.0 ± 0.0 | 3.0 ± 0.0 | 3.0 ± 0.0 | 2.8 ± 0.4 |
| - Limb Tone | 4.0 ± 0.0 | 4.0 ± 0.0 | 3.8 ± 0.5 | 3.5 ± 0.8 |
| Neurosensory Assessment | | | | |
| - Sensation Touch | 2.2 ± 0.8 | 2.5 ± 0.6 | 1.6 ± 0.7* | 1.6 ± 0.8* |
| - Suck Swallow | 2.4 ± 0.5 | 2.6 ± 0.5 | 2.5 ± 0.5 | 2.1 ± 0.3 |
| - Head Turning | 3.0 ± 0.0 | 3.0 ± 0.0 | 2.5 ± 0.5 | 2.1 ± 0.7 |
| - Odour Aversion | 2.8 ± 0.5 | 2.7 ± 0.5 | 2.0 ± 0.8* | 2.1 ± 0.8* |
| - Pain Sensation | 2.5 ± 0.5 | 1.6 ± 0.5* | 1.9 ± 0.6 | 1.7 ± 0.8* |

**S2 Table. Postnatal day 1 evaluation, PND1.** PCA31d n=11, PCA30d n= 11, PCA29d n= 8 and PCA28d n = 11. Data displayed as mean and SD with significance compared to the term birth group indicated as * 0.05 ≥ p > 0.01; ** 0.01 ≥ p > 0.001; ***p < 0.001.
