# Supplementary material for: Earlier preterm birth is associated with a worse neurocognitive outcome in a rabbit model
Source: PLoS One. 2021 Jan 27;16(1):e0246008. doi: 10.1371/journal.pone.0246008 (PMC7840009; doi:10.1371/journal.pone.0246008)
Supplement: S3 Table — PCA31d n = 11, PCA30d n = 11, PCA29d n = 7 and PCA28d n = 10. Data displayed as mean and SD with significance compared to the term birth group indicated as * 0.05 ≥ p > 0.01; ** 0.01 ≥ p > 0.001; ***p < 0.001. (DOCX) [file pone.0246008.s003.docx]

|  | Term birth  PCA31 | Preterm  PCA30d | Preterm  PCA29d | Preterm  PCA28d |
| --- | --- | --- | --- | --- |
| Sample phase interactions (n)   - 4 weeks - 8 weeks | 10.1 ± 2.5  9.3 ± 1.4 | 6.5 ± 2.3*  8.5 ± 2.2 | 4.6 ± 2.6**  8.5 ± 3.1 | 7.3 ± 2.9  7.6 ± 2.4 |
| Testing phase interactions (n)   - 4 weeks - 8 weeks | 5.5 ± 2.5  6.2 ± 2.5 | 6.6 ± 2.3  7.3 ± 3.1 | 5.1 ± 2.3  7.0 ± 3.1 | 4.3 ± 2.3  6.0 ± 3.7 |
| Novel object interactions (n)   - 4 weeks - 8 weeks | 3.8 ± 1.4  4.0 ± 1.2 | 4.4 ± 1.2  4.5 ± 2.1 | 2.9 ± 1.4  3.8 ± 1.4 | 2.1 ± 1.1  2.8 ± 1.8 |
| Discriminatory index   - 4 weeks - 8 weeks | 45.2 ± 29.8  34.5 ± 28.1 | 37.2 ± 25.6  29.3 ± 27.9 | 11.1 ± 14.4**  15.9 ± 21.5* | 1.4 ± 28.1*  -10.0 ± 34.5* |

**S3 Table. NORT - Novel object recognition test at 4 and 8 weeks of corrected postnatal age.** PCA31d n=11, PCA30d n= 11, PCA29d n= 7 and PCA28d n = 10. Data displayed as mean and SD with significance compared to the term birth group indicated as * 0.05 ≥ p > 0.01; ** 0.01 ≥ p > 0.001; ***p < 0.001.
