# Supplementary material for: Earlier preterm birth is associated with a worse neurocognitive outcome in a rabbit model
Source: PLoS One. 2021 Jan 27;16(1):e0246008. doi: 10.1371/journal.pone.0246008 (PMC7840009; doi:10.1371/journal.pone.0246008)
Supplement: S1 File — (DOCX) [file pone.0246008.s004.docx]

**Neurobehavioral Examination:** **PN +1 Evaluation**

For each animal, the testing will be videotaped and scored by two blinded observers. The kittens will be evaluated inside the incubator or in a designated space close to their pen with auditory and olfactory contamination kept to a minimum. Before handling they remained undisturbed in the assessment area for a 1-2min adaptation period.

- Cranial nerves were assessed by testing smell (olfaction was tested by recording time to aversive response to a cotton swab soaked with pure ethanol/peppermint water), sucking, and swallowing (by introduction of formula into the kittens’ mouth with a plastic pipette), and head turn to feeding. The responses will be graded on a scale of 0 to 3, 0 being the worst response and 3 the best response.
- Motor examination included tone, motor activity, and locomotion, righting reflex, and gait. Limb tone will be assessed by active flexion and extension of forelegs and hind legs (Ashworth scale).[1] The righting reflex will be assessed when the kittens were placed on their backs and the number of times turned prone within 2s. This was assessed 5 consecutive times. Gait will be examined based on a modification by Georgiadis et al.[2] Locomotion was assessed as described by Kannan et al.[3]
- Sensory examination is limited to touch on the face, touching the face with cotton swab or soft brush on both sides, and pain response by mild pin prick on the hind limb.
- To determine whether the preterm animals displayed adverse physiological consequences that might interfere with neurobehavioral testing, heart rate and rectal temperature are measured in a subgroup of randomly selected kittens at each evaluation.

| **Rabbit kit: Early neurobehavioral assessment PN+1d Date of evaluation:** \|__\|__\| \|__\|__\| \|__\|__\| | | | | | | | | | | | |
| --- | --- | --- | --- | --- | --- | --- | --- | --- | --- | --- | --- |
| **Rabbit ID:** \|__\|__\|__\|__\| | | | | **Weight (g):** | | | **PN day: +** | | **Video ID:** | | |
| During adaptation: \|__\| Sleepy \|__\| Awake Some activity \|__\| Active and exploring | | | | | | | | | | | |
| **NEUROMOTORIC** | | | | | | | **NEUROSENSORY** | | | | |
| **A. Posture** | | | | | | | **G. Surface Righting Reflex (5 attempts)** [2] | | | | |
| **0** | Lays supine | | | | | |  | no. of times turned to prone position within 2 sec seconds when placed in supine position | | | |
| **1** | Lays on side/minimal movement | | | | | |  |  |  |  |  |
| **2** | Cannot maintain prone position (roll over > 50% time) | | | | | | **H. Sensation - Facial/Whisker touch response** | | | | |
| **3** | Prone position with legs coiled (roll over < 50% time) | | | | | | **0** | No Response | | | |
| **B. Gait** [2] | | | | | | | **1** | Subtle (none directed reaction after ≥ 3 stimuli) | | | |
| **0** | Not able to move/uses whole body to move | | | | | | **2** | Mild (slow or directed response after 2 stimuli) | | | |
| **1** | Crawls, trunk touching ground, unstable > 50% time | | | | | | **3** | Immediate avoidance or brushes side of head | | | |
| **2** | Walks, trunk low/touching ground, unstable <50% time | | | | | | **I. Sucking and swallowing** | | | | |
| **3** | Walks but can’t propel body using hind legs synchronously | | | | | | **0** | No movement of jaw, milks dribbles out completely | | | |
| **4** | Walks, runs, and hops (synchronous) and coordinated | | | | | | **1** | Some jaw/neck movement, most milk dribbles out | | | |
| **C. Locomotion**[3] [all criteria must be met] | | | | | | | **2** | Definite suck and swallow, some milk in nose | | | |
| **0** | | Not able to move/uses whole body to move | | | | | **3** | Good suck and swallow, no milk in nose | | | |
| **1** | | Some movement, single steps, slight hops, <15cm | | | | | **J. Head turning during feeding** | | | | |
| **2** | | Good ROM, 2-3 continuous steps, 1-2 hops, 15-45cm | | | | | **0** | No movement | | | |
| **3** | | Entire ROM, >3 continuous steps, >3 hops, >45cm | | | | | **1** | Slow, occasional movement of head | | | |
|  | | Maximum number of continuous steps | | | | | **2** | Distinct movement of only head | | | |
|  | | Total distance travelled (cm) | | | | | **3** | Rapid forceful movements of head and body | | | |
|  | | Total number of squares crossed (n) | | | | | **K. Odour Aversion Test** | | Ethanol | Peppermint | |
|  | |  | | | | | **0** | No Response (>20s) |  |  | |
| **D. Motor Activity** | | | Head | | Fore limbs | Hind limbs | **1** | Subtle (6-20se) |  |  |  |
| **0** No Movement | | |  | |  |  | **2** | Low (2-5sec) |  |  |  |
| **1** Slight Movement | | |  | |  |  | **3** | Rapid/Normal (<2sec) |  |  |  |
| **2** Distinct Movement | | |  | |  |  | **L. Pain sensation – response to pin prick on limbs** | | | |  |
| **3** Rapid/Purposeful Movement | | |  | |  |  | **0** | No Response within 20 seconds with >3 pricks | | |  |
| **E. Duration of activity during observation** | | | | | | | **1** | Subtle (>2seconds, response with >2 pricks) | | | |
| **0** | | No movement | | | | | **2** | Mild (<2 seconds, rapid response with >1prick) | | | |
| **1** | | Active for < 20 seconds | | | | | **3** | Rapid/immediate, repetitive movement | | | |
| **2** | | Active for 20-40 seconds | | | | |  | | | | |
| **3** | | Active for > 40 seconds | | | | |  | | | | |
| **F. Limb Tone (Hind limbs)** [1] | | | | | | |  |  |  |  |  |
| **0** | | Limb rigid in flexion or extension | | | | |  |  |  |  |  |
| **1** | | Tone considerably increased; passive movement difficult | | | | |  |  |  |  |  |
| **2** | | Marked increase in tone but limb is easily flexed | | | | |  |  |  |  |  |
| **3** | | Slight increase in tone when limb is moved | | | | |  |  |  |  |  |
| **4** | | No increase in tone | | | | |  |  |  |  |  |
|  | | | | | | |  |  |  |  |  |

*# Performed on designated 25 x 25 cm area divided into 5 x 5 cm blocks over a time period of 90sec.*

**Neurobehavioral assessment** - **Open Field Test:**

Evaluation of emotional reactivity and anxiety, exploratory- and non-exploratory locomotion will be assessed. Rabbits will be handled daily and habituated in the test room before the test. For habituation, the animals will be placed in the evaluation room for 30 min. The OFT was designed and used in accordance with the procedure previously described.[4,5]

A pen with 80cm-high polyvinyl chloride walls and a rubber floor divided into nine numbered squares will be used. The total duration of the test will be 5 minutes per animal: each rabbit will be placed in a starting box (25 x 25 x 80cm) for one minute. After opening the starting box if the animal fails to start exploration after one minute, it was gently pushed into the pen.

|  |  | 80cm |  |  |
| --- | --- | --- | --- | --- |
|  |  |  |  | 80cm |
|  | 20cm | Central |  |  |
|  | Starting box | 20cm |  |  |

|  |  | 150cm |  |  |
| --- | --- | --- | --- | --- |
|  |  |  |  | 150cm |
|  | 25cm | Central |  |  |
|  | Starting box | 25cm |  |  |

The following behaviours were considered:(7–9)

- latency of leaving the starting point (seconds),
- Total and central displacements (number of squares crossed in the pen and in the middle square) including total time spent in central and peripheral squares (seconds)
- Movement (time spent moving with fore and hind legs among squares) total distance travelled, mean speed, mobile time
- Running (time spent running among squares)
- Escape attempts (number of rapid runs toward the corners of the pen)
- Exploration (the time spent moving with forelegs or standing while sniffing and looking around inside the same square)
- Hops (the number of times the rabbit completely displaced its body by a hop)
- Standing still (time spent still with fore and hind legs not stretched and, on the ground)
- Resting (time spent inactive, with the body touching the floor and fore and/or hind legs stretched on the ground)
- Rearing (number of times the rabbit upheaves on its hind legs)
- Self-grooming (the time spent in self-grooming); digging (the time spent in digging inside the pen), biting (time spent in biting elements of the pen)
- Defecation (number of times the rabbit defecated); and urination (number of times the rabbit urinated).

The room was insulated from sound and with full overhead illumination. The area was cleaned for urine and faeces after each test. To minimize interference due to human contact, each session was video-taped and later evaluated by two blinded observers.

| **Rabbit: Open Field Test Date of evaluation:** \|__\|__\| \|__\|__\| \|__\|__\| | | | | | | |
| --- | --- | --- | --- | --- | --- | --- |
| **Rabbit ID:** \|__\|__\|__\|__\| | **Weight (g):** | **PN day: +** | | | **Video ID:** | |
| **In the starting box: (max 60sec)** | | |  | **Comment** | | |
| Entered the pen field (Yes/No) | | |  |  | | |
| Duration before entering the pen (seconds) | | |  |  | | |
| Number of movements in the starting box (number) | | |  |  | | |
| **Global activity in the open field: (5/10min)** | | |  |  | |  |
| Total displacement (number of squares crossed) | | |  |  | | |
| Total movement (total time moving in seconds) | | |  |  | | |
| Central displacement (number of times central squares crossed) | | |  |  | | |
| Time spent in central squares (seconds) | | |  |  | | |
| Total distance travelled () Mean speed () | | |  |  | | |
| **Behavioural activity in the open flied:** | | |  |  | |  |
| Escape attempts | | |  |  | | |
| Exploration (seconds) | | |  |  | | |
| Running (seconds) | | |  |  | | |
| Standing Still (seconds) | | |  |  | | |
| Resting (seconds) | | |  |  | | |
| Time spent in corners (seconds) | | |  |  | | |
| Rearing (seconds, number of episodes) | | |  |  | | |
| Self-grooming (seconds) | | |  |  | | |
| Digging (seconds, number of episodes) | | |  |  | | |
| Biting (seconds, number of episodes) | | |  |  | | |
| Hops (total number) | | |  |  | | |
| Urinating (number of episodes) | | |  |  | | |
| Defecating (number of episodes) | | |  |  | | |
|  | | |  |  | | |

*# Performed on designated 80 x 80 cm area divided into 20 x 20 cm blocks over a time period of 5min.*

- **Escape attempts**: number of rapid runs toward the corners of the pen.
- **Exploration**: Time spent moving with forelegs or standing while sniffing and looking around inside the same square, head movements indicating investigation of the environment. Exploration is an independent category, but it can occur together with standing stretched, moving forelegs and walking.
- **Standing still:** Time spent still with fore and hind legs not stretched and on the ground.
- **Resting**: Time spent inactive, with the body touching the floor and fore and/or hind legs stretched on the ground.
- **Rearing:** Number of times the rabbit upheaves on its hind legs, the rabbit stands on its heels.
- **Self-grooming:** Time spent in self-grooming including licking, scratching or nibbling of the body.
- **Digging:** Time spent in digging inside the pen with forelegs scratching on the floor or wall.
- **Biting**: Time spent in biting elements of the pen.
- **Hopping**: the forelegs are moved alternately; the hind legs are moved synchronously. The number of times the rabbit completely displaced its body by a hop.
- **Bounding**: moving upwards/forwards with all feet off the floor. Can be accompanied by sideward/upward swinging of the rear.

**Neurobehavioral assessment – Novel Object Recognition Test:**

Assesses behavioural response to novelty, object-directed exploration to assess recognition memory and declarative memory through hippocampus-dependent memory consolidation. The Novel Object Recognition Task (NORT) will be performed in the OFT pen and was adapted from the original description [7] including some modifications in the stimulus used. Instead of using visual stimulus, odour-based stimulus will be used by means of placing pieces of fruit (apple or orange) inside perforated plastic boxes, since olfactory sensitivity is highly developed in rabbits.[8] This is in agreement with the notion that the type of stimulus presented must be one in which the sensory perception of the species chosen is adequate.

- ‘Sampling phase’ First, two boxes containing the same odour-based stimuli (apple) will be presented to the animal for 5 minutes. This constituted a familiarization phase.
- ‘Retention phase’ the rabbit will then be returned to its cage for a 15-minute retention interval.
- ‘Testing / Choice phase’ Then, one of the objects will be removed and replaced by a novel stimulus (orange). Exploration of the object was considered when the rabbit showed sniffing, touching and having moving vibrissae while directing the nose towards the object at a distance of less than 1 cm.

Cumulative time (seconds) exploring each object in the two sessions will be recorded (right and left objects in the Familiarization phase, whereas novel and the familiar objects in the Testing phase). Finally, the discrimination index (DI), which represents the ability to discriminate the novel from the familiar object, will be calculated as follows:

*DI =*$\frac{(Novel Object Exploration Time - Familiar Object Exploration Time)}{(Novel Object Exploration Time + Familiar Object Exploration Time)}$

Learning criteria was considered when the DI was above 0.

Animals that did not explore the familiar object at least once in the Testing phase or did not explore any of the objects in the Familiarization phase will be excluded from the analysis, as previously suggested.[9]

| **Rabbit: Novel Object Recognition Test Date of evaluation:** \|__\|__\| \|__\|__\| \|__\|__\| | | | | | | |
| --- | --- | --- | --- | --- | --- | --- |
| **Rabbit ID:** \|__\|__\|__\|__\| | **Weight (g):** | **PN day: +** | | |  | |
| Objects in sample phase: | | Objects in Testing phase: L or R | | | | |
| **Sample Phase 5 / 10 min** | | | Video ID: | | | |
| Number of interactions with objects/stimuli (number for Lt and Rt) | | | Lt | Rt | | Total |
| Total time of interaction with objects/stimuli (second for Lt and Rt) | | | Lt | Rt | | Total |
|  | | |  |  | |  |
| **Retention phase (10/30/45/60 min)** | | |  | | | |
| **Testing Phase 5 / 10 min** | | | Video ID: | | | |
| Number of interactions with **FIMILIAR** objects/stimuli (number) | | |  | | | |
| Total time of interaction with **FIMILIAR** objects/stimuli (seconds) | | |  | | | |
| Number of interactions with **NOVEL** objects/stimuli (number) | | |  | | | |
| Total time of interaction with **NOVEL** objects/stimuli (seconds) | | |  | | | |
| **DI = (*T*_N_ − *T*_F_) / (*T*_N_ + *T*_F_)** | | |  | | | |
|  | | |  | | | |

- **Habituation**: Animals were placed in the open pen for 30 min without any objects present.
- **Sampling phase**: Two boxes containing the same odour-based stimuli (apple) were presented to the animal for 5 minutes.
- **Retention phase:** Rabbit was then returned to its cage for a 10-30minute retention interval.
- **Testing / Choice phase**: One of the objects was removed and replaced by a novel stimulus (orange) and the animal was again placed in the area with the novel and familiar objects for 5 minutes more.

**Neurobehavioral assessment – T-maze Spontaneous Alteration Test:**

To assess spatial learning and memory. The T-maze spontaneous alternation test will be used as published methods for rodents [10] and modified for rabbits [11]. Rabbits will be tested for three consecutive days, four trials per animal per day. The behavioural assessments will be conducted blinded to group allocation. The first two trials will be in the morning and the second two trials in the afternoon, with an interval of at least 4–5 h.

For each trial, the rabbits will be placed in the start area in an in-house-made T-maze (goal arms, 90×20×80 cm; start arm, 90×20×80 cm) and confined for 30 sec before they will be allowed to choose a goal arm. The rabbits will be considered to have made a choice when they placed all four paws into one arm. Thereafter, the rabbits will be allowed to explore the chosen goal arm for 30-60 sec. Then, they will be removed from the goal arm and placed back in the cage before being given the next trial. The inter-trial interval will be 15 min. The number of alternations divided by the total number of choices will be calculated and averaged.

*Spontaneous alteration percent =* $\frac{Number of alterating choices}{Total number of choices}$ *x 100*

| 20cm | **Goal Arm** |  | Sliding Door |  |  |  | Sliding Door |  | **Goal Arm** |
| --- | --- | --- | --- | --- | --- | --- | --- | --- | --- |
|  |  | 90cm |  | |  |  | |  |  |
|  |  |  | 90cm | | Sliding Door |  | |  |  |
|  |  |  |  | | **Starting Box** | 25cm | |  |  |
|  |  |  |  | | 20cm |  | |  |  |

| **Rabbit: T-Maze Test Date of evaluation:** \|__\|__\| \|__\|__\| \|__\|__\| | | | | | |
| --- | --- | --- | --- | --- | --- |
| **Rabbit ID:** \|__\|__\|__\|__\| | **Weight (g) :** | | **PN day: +** |  | |
| **Day 1 AM** | | | Video ID | | |
| Sample Run Arm Choice – L (left), R (Right), F (Failed) | |  | Test Run Arm Choice – L (left), R (Right), F (Failed) | |  |
|  | |  |  | |  |
| **Day 1 PM** | | | Video ID | | |
| Sample Run Arm Choice – L (left), R (Right), F (Failed) | |  | Test Run Arm Choice – L (left), R (Right), F (Failed) | |  |
|  | |  |  | |  |
| **Day 2 AM** | | | Video ID | | |
| Sample Run Arm Choice – L (left), R (Right), F (Failed) | |  | Test Run Arm Choice – L (left), R (Right), F (Failed) | |  |
|  | |  |  | |  |
| **Day 2 PM** | | | Video ID | | |
| Sample Run Arm Choice – L (left), R (Right), F (Failed) | |  | Test Run Arm Choice – L (left), R (Right), F (Failed) | |  |
|  | |  |  | |  |
| **Day 3 AM** | | | Video ID | | |
| Sample Run Arm Choice – L (left), R (Right), F (Failed) | |  | Test Run Arm Choice – L (left), R (Right), F (Failed) | |  |
|  | |  |  | |  |
| **Day 3 PM** | | | Video ID | | |
| Sample Run Arm Choice – L (left), R (Right), F (Failed) | |  | Test Run Arm Choice – L (left), R (Right), F (Failed) | |  |
|  | |  |  | |  |
| **Total Alterations (n)** | |  | **Alterations % (Total Alterations/6 x 100)** | |  |

1. Damiano DL, Quinlivan JM, Owen BF, Payne P, Nelson KC, Abel MF. What does the Ashworth scale really measure and are instrumented measures more valid and precise? Dev Med Child Neurol. 2002;44: 112–118.

2. Georgiadis P, Xu H, Chua C, Hu F, Collins L, Huynh C, et al. Characterization of acute brain injuries and neurobehavioral profiles in a rabbit model of germinal matrix hemorrhage. Stroke J Cereb Circ. 2008;39: 3378–3388. doi:10.1161/STROKEAHA.107.510883

3. Kannan S, Dai H, Navath RS, Balakrishnan B, Jyoti A, Janisse J, et al. Dendrimer-Based Postnatal Therapy for Neuroinflammation and Cerebral Palsy in a Rabbit Model. Sci Transl Med. 2012;4: 130ra46. doi:10.1126/scitranslmed.3003162

4. Ferrante V, Verga M, Canali E, Mattiello S. Rabbits Kept in Cages and in Floor Pens: Reactions in the Open Field-Test. J Appl Rabbit Res. 1992;15: 700–700.

5. Meijsser FM, Kersten AMP, Wiepkema PR, Metz JHM. An analysis of the open-field performance of sub-adult rabbits. Appl Anim Behav Sci. 1989;24: 147–155. doi:10.1016/0168-1591(89)90042-7

6. Trocino A, Filiou E, Tazzoli M, Bertotto D, Negrato E, Xiccato G. Behaviour and welfare of growing rabbits housed in cages and pens. Livest Sci. 2014;167: 305–314. doi:10.1016/j.livsci.2014.05.035

7. Ennaceur A, Delacour J. A new one-trial test for neurobiological studies of memory in rats. 1: Behavioral data. Behav Brain Res. 1988;31: 47–59.

8. Ennaceur A. One-trial object recognition in rats and mice: methodological and theoretical issues. Behav Brain Res. 2010;215: 244–254. doi:10.1016/j.bbr.2009.12.036

9. de Bruin N, Pouzet B. Beneficial effects of galantamine on performance in the object recognition task in Swiss mice: deficits induced by scopolamine and by prolonging the retention interval. Pharmacol Biochem Behav. 2006;85: 253–260. doi:10.1016/j.pbb.2006.08.007

10. Deacon RMJ, Rawlins JNP. T-maze alternation in the rodent. Nat Protoc. 2006;1: 7–12. doi:10.1038/nprot.2006.2

11. Zhang Z, Saraswati M, Koehler RC, Robertson C, Kannan S. A New Rabbit Model of Pediatric Traumatic Brain Injury. J Neurotrauma. 2015;32: 1369–1379. doi:10.1089/neu.2014.3701
